# Photoinitiated Polymerization‐Induced Self‐Assembly (Photo‐PISA): New Insights and Opportunities

**DOI:** 10.1002/advs.201700137

**Published:** 2017-05-30

**Authors:** Jonathan Yeow, Cyrille Boyer

**Affiliations:** ^1^ School of Chemical Engineering Centre for Advanced Macromolecular Design (CAMD) and Australian Centre for Nanomedicine (ACN) UNSW Sydney Sydney NSW 2052 Australia

**Keywords:** controlled/living radical polymerization, photopolymerization, polymerization‐induced self‐assembly, visible light, Photo‐PISA

## Abstract

The polymerization‐induced self‐assembly (PISA) process is a useful synthetic tool for the efficient synthesis of polymeric nanoparticles of different morphologies. Recently, studies on visible light initiated PISA processes have offered a number of key research opportunities that are not readily accessible using traditional thermally initiated systems. For example, visible light mediated PISA (Photo‐PISA) enables a high degree of control over the dispersion polymerization process by manipulation of the wavelength and intensity of incident light. In some cases, the final nanoparticle morphology of a single formulation can be modulated by simple manipulation of these externally controlled parameters. In addition, temporal (and in principle spatial) control over the Photo‐PISA process can be achieved in most cases. Exploitation of the mild room temperature polymerizations conditions can enable the encapsulation of thermally sensitive therapeutics to occur without compromising the polymerization rate and their activities. Finally, the Photo‐PISA process can enable further mechanistic insights into the morphological evolution of nanoparticle formation such as the effects of temperature on the self‐assembly process. The purpose of this mini‐review is therefore to examine some of these recent advances that have been made in Photo‐PISA processes, particularly in light of the specific advantages that may exist in comparison with conventional thermally initiated systems.

## 1 Introduction

In recent years, controlled/living radical polymerization (CLRP) techniques under surfactant free dispersion polymerization conditions have been used to synthesize in situ self‐assembled nanoparticles according to a process known as polymerization‐induced self‐assembly (PISA). This process involves the in situ production of self‐assembled polymeric nanoparticles by the chain extension of a living solvophilic polymer precursor with a monomer which forms a solvophobic polymer. Such a process can take place under dispersion or emulsion polymerization conditions and has been successfully demonstrated in a range of different solvent systems such as water,^1^ alcohols,^2^ alkanes,^3^ ethylene glycol,^4^ supercritical carbon dioxide^5^ and even ionic liquids.[[qv: 1q]] Interest in the PISA process for the synthesis of polymeric nanoparticles has been primarily driven by interest in the use of these particles in a broad range of applications such as carriers for drug/therapeutic delivery[[qv: 1r,2e,6]] or imaging agents,[[qv: 2i,7]] inorganic particle dispersants/templates,[[qv: 2j,8]] catalytic nanoreactors,^9^ cryoprotective gels for living cells,^10^ viscosity modifiers for lubricants,[[qv: 3c]] Pickering emulsifiers^11^ and stimuli responsive smart nanomaterials.^12^

Whilst conventional self‐assembly techniques are typically performed under relatively dilute conditions (<1 wt%), the PISA technique (under dispersion or emulsion conditions) allows for the production of nanomaterials under relatively concentrated conditions (10–50 wt%). This approach is therefore less sensitive to issues of scale. Furthermore, the PISA technique allows for more facile access to a range of higher order morphologies in a more reproducible manner compared to conventional self‐assembly. It should be noted that currently, the vast majority of PISA polymerizations are conducted under reversible addition‐fragmentation chain transfer (RAFT) dispersion or emulsion polymerization conditions using thermally initiated radical sources such as azo‐initiators. However, there are some reports on the use of other techniques for initiating a PISA process including atom transfer radical polymerization (ATRP),[[qv: 6d,13]] nitroxide mediated polymerization (NMP),^14^ ring opening metathesis polymerization (ROMP),^15^ cobalt mediated polymerization (CMP)^16^ and organotellurium‐mediated radical polymerization (TERP).^17^

Some of the earliest works by Hawkett et al. using RAFT emulsion polymerization demonstrated that polymer latexes could be synthesized from in situ self‐assembled micelles although a controlled monomer feed was required to prevent monomer droplet formation.^18^ Subsequent work has since shown that controlled RAFT emulsion polymerization in batch is also possible although compared to RAFT dispersion polymerization there have been limited reports of particle evolution beyond spherical particles (S).[[qv: 1c,14b,19]] Some of the earliest investigations into the PISA process occurring under RAFT dispersion conditions were demonstrated by the groups of Pan^20^ and Armes,^21^ primarily to address some of the shortcomings of conventional self‐assembly processes. RAFT dispersion polymerizations are commonly used to study complex self‐assembly processes with a focus on the formation of higher order morphologies (beyond spheres), such as worm‐like micelles (WLM) and vesicles (V).

A number of parameters are known to strongly influence the morphology of nanoparticles synthesized using a PISA approach. For example, higher order morphologies are generally favoured by increasing the length of the solvophobic block, increasing monomer concentration, using relatively low molecular weight macroRAFT agents and improving the mobility of the solvophobic polymer chains. Generally, however, morphological transitions can be difficult to predict owing to a broad number of kinetic and thermodynamic factors which may influence the necessary rearrangement of polymer chains. For example, most RAFT emulsion (and some dispersion) polymerizations form exclusively spherical particles that only increase in diameter during the polymerization and do not rearrange into higher order morphologies as expected from an increased packing parameter. This phenomenon is generally attributed to the limited mobility of the core forming polymer chains which results in so‐called kinetically trapped spheres.^22^ The difficulty in predicting nanoparticle morphology is further complicated by additional factors such as electrostatic interactions,^23^ monomer/polymer solubility in the solvent,[[qv: 2d,22b]] degree of polymerization control (i.e. polymer dispersity)^24^ and the compatibility of the two polymer blocks.^25^ In addition, extrinsic experimental conditions such as the polymerization temperature[[qv: 6b,14c,26]] and cooling procedure[[qv: 3g]] can also have a significant impact on the morphology of the synthesized nanoparticles. For example, a few studies have indicated that in some cases the morphology formed at the polymerization temperature (without crosslinking) may be different to that formed upon cooling to room temperature which may affect the reproducibility of the syntheses.^27^

In order to improve the general reproducibility of the PISA technique, it is possible to generate phase diagrams as a means to assist in the targeting of different nanoparticle morphologies.[[qv: 1s,3b]] Typically, these PISA based phase diagrams are generated by varying the target degree of polymerization (DP) (y‐axis) against the total solids content (x‐axis) and observing the predominant nanoparticle morphology (or morphologies for mixed phases) by TEM. For example, a comprehensive study by Blanazs et al. demonstrated that a phase diagram generated using a relatively long solvophilic polymer was exclusively populated by spherical morphologies.[[qv: 1s]] In contrast, the use of a shorter stabilizing polymer under a similar range of reaction conditions allowed the formation of higher order morphologies. These phase diagrams therefore provide a useful insight into the qualitative trends of the RAFT dispersion process by acting as a “roadmap” for nanoparticle synthesis. Several excellent reviews on the state of the current PISA literature are available elsewhere.[[qv: 1i,2a,3a,20,21,22,27,28]]

## 2 Light Mediated RAFT Polymerization

Historically, the use of thermally activated radical sources has been the most common method to initiate CLRP techniques such as RAFT. However, in the last few years, there has been intense interest in using alternate forms of initiation to drive CLRP polymerizations. For example, several alternative techniques have recently been reported for initiating a RAFT‐type polymerization such as (photo)redox catalysts,^29^ enzymes,^30^ organic acids (radical^31^ and cationic^32^ mechanisms), and direct activation of the RAFT agent itself.^33^ In particular, the use of electromagnetic radiation to initiate RAFT polymerization has been extensively explored and has been demonstrated with a broad range of wavelengths including the gamma,^34^ ultraviolet (UV),[[qv: 33c,35]] visible,[[qv: 29a,36]] near infrared (NIR)^37^ and microwave^38^ regions of the spectrum.

As an alternative to other initiation methods (thermal, gamma etc.), the use of visible or NIR light for controlling a CLRP is particularly attractive owing to their relatively low energy requirements (particularly the use of light‐emitting diodes (LEDs)), less side reactions and potential to exert temporal and spatial control over the polymerization.^39^ In addition, since these polymerizations can occur at ambient temperatures, the facile conditions are more favourable for polymerization in the presence of proteins, DNA and other temperature sensitive moieties.[[qv: 39d,40]] For example, the mild conditions associated with light mediated polymerization allow for polymerizations to occur in the presence of thermally incompatible moieties.^41^ Whilst low temperature thermal initiators can also be used for such applications,[[qv: 40c,42]] they are generally more difficult to handle and should be removed prior to storage of these samples due to non‐negligible degradation that can occur at ambient temperature which might result in changes to morphology. Furthermore, light mediated polymerizations can be readily turned ON/OFF, facilitating the isolation of different nanoparticle morphologies during the polymerization in contrast to thermal polymerizations.

Although light mediated CLRP techniques based on ATRP,^43^ NMP,^44^ RAFT[[qv: 29a,36d,45]] and other^46^ CLRP techniques have been extensively developed over the last few years, only RAFT photopolymerization has been successfully implemented in a PISA process for the synthesis of a broad range of nanoparticle morphologies. A significant body of work by Yoshida has reported the use of UV light to initiate the NMP of methyl methacrylate under dispersion polymerization conditions, however only micron sized particles with broad size/morphology distributions were typically achieved.[[qv: 44e,47]] For the purposes of this review, visible light mediated photopolymerizations are classified as those initiated/controlled using light sources emitting at wavelengths greater than 400 nm (or with suitable UV light filters).

## 3 UV Initiated RAFT Dispersion Polymerization

Some early works demonstrated the use of UV light to initiate conventional free radical dispersion polymerizations. For example, Chen et al. demonstrated that microspheres could be obtained at relatively rapid rates when using UV activated photoinitiators (Darocur 1173) in the presence of poly(*N*‐vinylpyrrolidone) (PVP) as a stabilizer.^48^ However, the addition of RAFT agent can result in significant deviations from ideal dispersion polymerization behaviour. For example, Choe and coworkers used the photolysis of a dithiobenzoate RAFT agent under a 1 kW UV lamp to initiate the dispersion polymerization of styrene in ethanol and in the presence of PVP as a stabilizer.^49^ Under these conditions, some aspects of living behaviour were observed such as an increase in molecular weight with conversion however the polymer dispersities were typically close to 2.0. Furthermore, the presence of the RAFT agent resulted in a relatively long nucleation period and hence quite broad particle size distributions compared to those achievable without RAFT agent. Generally, as the RAFT concentration increased, there was a decrease in polymer dispersity but a concomitant increase in particle dispersity. Youk and coworkers also reported difficulty in simultaneously controlling both the particle size distribution and molecular weight distribution using the RAFT photolysis approach.^50^ Later, Tan et al. demonstrated that in a dispersion polymerization initiated by a conventional photoinitiator, the addition of RAFT agent could slow the nucleation process allowing for the stabilizer to mediate the nucleation process more uniformly yielding narrowly distributed microspheres.^51^ This batch process enabled the formation of more uniform microspheres without the need for the two‐stage process suggested by Winnik and coworkers in thermally initiated dispersion polymerization systems.^52^ However, the degree of RAFT control was limited by the relatively high photoinitiator to RAFT ratios employed. Nonetheless, photoinitiation has been shown to be a suitable method for synthesising “living” microspheres (with varying degrees of livingness) that can be further modified by a “grafting from” approach. Furthermore, the use of functional macromolecular RAFT agents (macroRAFT) as a dispersion polymerization stabilizer has enabled the production of uniform surface functional microspheres for applications such as multiplexed bioassays.^53^ In order to improve the degree of RAFT control over the dispersion process, Chen and coworkers employed a lower ratio of initiator to macroRAFT agent.^54^ Under UV light, the dispersion polymerization of styrene yielded spherical self‐assembled micelles according to a PISA approach rather than microspheres typically obtained in a conventional dispersion polymerization. These early works demonstrated the feasibility of using light to initiate a PISA process although only spherical nanoparticles could be obtained. The remainder of this review will focus on *visible light* mediated PISA approaches, particularly when used in the synthesis of nanoparticles with well‐defined morphologies.

## 4 Visible Light Initiated PISA (Photo‐PISA)

**Figure 1:**
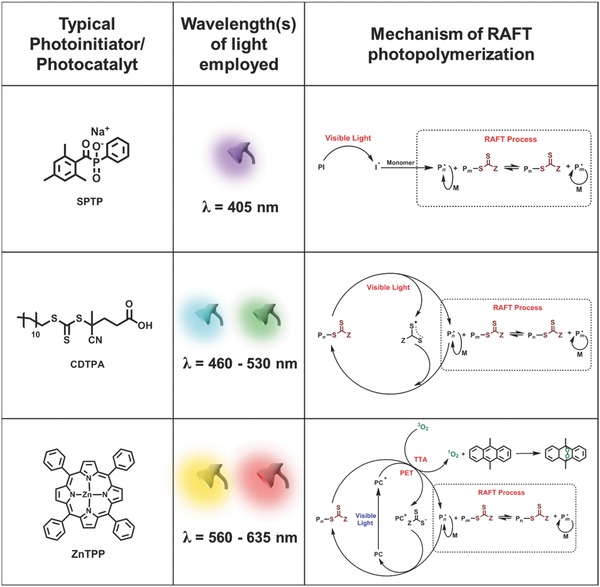
Different mechanisms for initiating PISA polymerizations under visible light: (top row) photoinitiator (PI), (middle row) photoiniferter and (bottom row) photocatalyst (PC) approaches.

In this section, we will highlight some of the recent advances in visible light initiated PISA which are categorized according to the mechanism of radical production (**Figure** **1**).

### 4.1 Visible Light Photoinitiators for Conducting a PISA Process

Photoinitiators are light absorbing compounds which under UV or visible light generate species (such as reactive radicals or ions) capable of initiating polymerization. For the purposes of this review, we will consider only photoinitiators that generate radical species for initiating radical polymerization of vinyl monomers. They are typically classified according to whether they generate radicals in a unimolecular homolytic cleavage reaction (Type I) or bimolecular reaction with a hydrogen donor such as an amine or thiol (Type II).^55^ Whilst the majority of industrially used photoinitiators (such as Irgacure^®^ compounds) absorb only in the UV region of the spectrum, there are a number of photoinitiators capable of generating radicals under visible light irradiation.[[qv: 39a,56]] In the presence of a suitable RAFT agent, photoinitiators have been used to synthesize well defined polymers under a range of both homogenous and heterogenous conditions.[[qv: 1r,35a,36a,40d,51a,53b,54,57]]

The first example of a visible light initiated Photo‐PISA process yielding self‐assembled nanoparticles was reported by Cai and co‐workers in 2015 using SPTP as a water soluble radical photoinitiator and a 400 W mercury lamp source (with a 400 nm filter).^58^ In this work, a poly(2‐hydroxypropyl methacrylamide) (PHPMAm) macroRAFT was chain extended with diacetone acrylamide (DAAm) in water under visible light irradiation at 25 °C. Fast polymerization rates were observed reaching high monomer conversions within one hour of irradiation whilst still maintaining reasonable control over the polymerization when targeting lower degrees of polymerization (Ð < 1.3). The insolubility of the poly(DAAm) block led to the in situ formation of broadly defined spherical nanoparticles stabilised by the PHPMAm block. Incorporation of *N*‐(2‐aminoethyl) acrylamide (AEAM) as a comonomer (under acidic conditions to prevent RAFT aminolysis) allowed for the installation of metal binding motifs via post‐polymerization modification. More recently, the same photopolymerization technique was used to synthesize an unusual collection of morphologies such as silk‐like films, ribbons, interlinked vesicles and nanotubes.^59^ These morphologies were strongly influenced by the ability of the DAAm units to hydrogen bond with one another which may explain why these supramolecular structures have not previously been observable when the polymerization was performed thermally at 70 °C. Interestingly, the copolymerization of a small ratio of AEAM enables tuning of the porosity of the as‐synthesized nanotubes and vesicles. By varying the pH, the degree of ionization of the AEAM primary amine could be varied and thereby affect the porosity of the vesicle membrane. These nanostructures are likely to have a number of applications as intelligent nano‐membranes in biological applications. The same group has also used this approach to demonstrate polyion complexation between a monomer‐polymer template pair for the synthesis of spherical and network nanostructures.^60^

**Figure 2:**
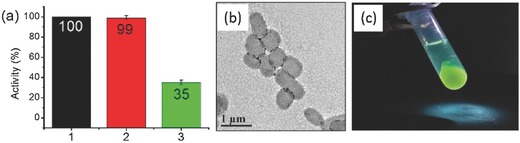
Encapsulation of BSA (without denaturation) into the lumen of vesicles under facile room temperature photopolymerization conditions. Reproduced with permission.[[qv: 1r]] Copyright 2015, American Chemical Society.

Zhang, Sumerlin and coworkers expanded upon this approach by employing violet LED light (λ = 405 nm) in conjunction with the photoinitiator, SPTP to polymerize 2‐hydroxypropyl methacrylate (HPMA) from a poly(ethylene glycol) (PEG) based macroRAFT agent. Under aqueous RAFT dispersion conditions, this Photo‐PISA approach led to a diverse set of morphologies with S, WLM and V (unilamellar and multilamellar) morphologies obtained by varying the target DP of HPMA and the total solids content.[[qv: 1r]] Using the same photoinitiator reported in Cai's work (SPTP), ultrafast kinetics were observed with quantitative monomer conversions achievable within 30 min at room temperature. In comparison, a typical aqueous RAFT dispersion initiated using the azoinitiator, 4,4′‐azobis(4‐cyanovaleric acid (ACVA) at 70 °C typically requires greater than 3 hours to achieve quantitative conversions.[[qv: 1a,61]] Importantly, the full range of nanoparticle morphologies was readily accessible and comprehensive experiments yielded the first report of a PISA phase diagram synthesized at room temperature. Interestingly, the phase diagram generated here for PEG_113_ *‐b‐*PHPMA differs qualitatively from the same block copolymer synthesis performed at 50 °C by Armes and coworkers. It is likely that this effect is due to either the varying polymerization rates and/or the differing polymerization temperatures affecting the degree of solvent/monomer partitioning. However, it should be noted that the difference in RAFT agent structure and fraction of residual unesterified PEG (typically < 5%) would not be completely negligible. The addition of silica nanoparticles (at 30% solids) to the initial polymerization mixture allowed for the in situ encapsulation of the inorganic particles into the lumen of the polymer vesicles. During this loading process, the added silica had no effect on the polymerization kinetics and unencapsulated silica could be removed by gentle centrifugation. This facile in situ loading process was further explored to load bovine serum albumin (BSA) as a model protein (**Figure** **2**). Importantly, the BSA maintained > 90% of its biological activity (as determined by a hydrolysis assay with 4‐nitrophenyl acetate) with free BSA removable via repeated centrifugation and redispersion. In contrast, when placed under thermally initiated PISA conditions at 70 °C, more than 60% of BSA activity was lost. This data demonstrates a significant advantage of these room temperature polymerization techniques due to their compatibility with biological species such as proteins. It should be noted that at the same time, Armes and coworkers also demonstrated this method of protein encapsulation, however, a low temperature thermal initiator (VA‐044 at 37 °C) in addition to a longer polymerization time was required in order to minimise protein denaturation.[[qv: 8b]]

The same group has also explored the use of other water soluble macroRAFT stabilizers such as those based on thermoresponsive poly(ethylene glycol) methacrylates.[[qv: 6b,57f]] Copolymerization of ethylene glycol based methacrylates can yield thermoresponsive polymers with well‐defined and tuneable LCST behaviour in water. The use of a stabilizer block with thermoresponsive behaviour is difficult to achieve under typical thermally initiated PISA conditions (70 °C), owing to a loss of particle stability close to the LCST. Interestingly, in this study the authors determined that the observed LCST of the thermoresponsive macroRAFT was further lowered in the presence of the HPMA monomer by as much as 29 °C and thereby prevented its controlled polymerization at 20 °C. However, by lowering the polymerization temperature to 15 °C (or lower) the dispersion polymerization proceeded with quantitative monomer conversion within 15 min of irradiation with a reasonable degree of control being maintained over the polymerization. This process is only possible due to the relative temperature insensitivity of radical production by SPTP under visible light. Importantly, the full range of nanoparticle morphologies (S, WLM, V) could be obtained even at the lowered polymerization temperature. Spherical nanoparticles were shown to retain the thermoresponsive behaviour of the stabilizing macroRAFT agent, and underwent a decrease in size from 85 nm to 61 nm as the temperature was increased above the LCST. DLS was used to demonstrate the stability of these particles at temperatures above the LCST however the morphology at different temperatures or the reversibility of this process was not reported. Nonetheless, this study demonstrates a key advantage of photopolymerization in performing dispersion polymerizations below room temperature.

**Figure 3:**
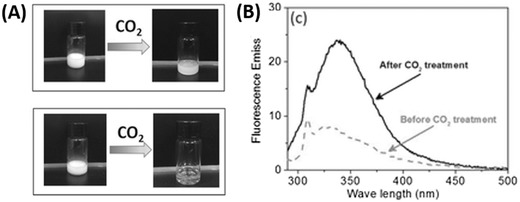
(A) PDMAEMA functionalized vesicles synthesized using a Photo‐PISA approach can be dissembled under application of carbon dioxide as a stimulus. (B) This process can be used to release lumen encapsulated BSA due to disassembly of the vesicular structure. Adapted with permission.^62^

Recently, the same group studied the effect of incorporating a minor fraction of the tertiary amine containing monomer, 2‐(dimethylamino)ethyl methacrylate (DMAEMA) into the HPMA core‐forming polymer block.^62^ Although significant deviations from living polymerization behaviour were observed (attributed to the amine groups acting as co‐initiators), the full range of nanoparticle morphologies could still be accessed as in previous studies. Importantly, the tertiary amine moieties within the core could be protonated by bubbling with carbon dioxide (due to the formation of carbonic acid). In general, treatment with CO_2_ resulted in a decrease in particle size or in some cases complete solvation of the polymer chains (**Figure** **3** **A**). Finally, the authors demonstrated the loading of BSA into vesicles under mild photopolymerization conditions and its subsequent release in the presence of dissolved CO_2_ (Figure 3B). This result is particularly promising for applications requiring the controlled release of encapsulated proteins under mild conditions. This photoinitiator approach has also been extended to the dispersion polymerization of isobornyl acrylate in an ethanol/water mixture (85/15 w/w).^63^ These polymerizations reached high conversion under violet light (405 nm, 0.5 mW cm^−2^) within 30 min due to the fast decomposition lifetime of TPO (compared to AIBN at 70 °C). Despite the ultrafast kinetics, the alcoholic dispersion polymerization was well‐controlled (Đ < 1.3) and the full range of nanoparticle morphologies could be accessed by varying the monomer concentration and target DP.

**Figure 4:**
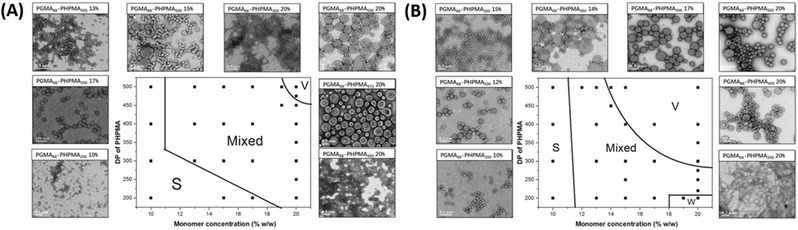
Phase diagrams of RAFT dispersion polymerization of HPMA initiated by SPTP at (A) 25 and (B) 70 °C. Adapted with permission.^65^ Copyright 2017, The Royal Society of Chemistry.

Apart from the use of redox initiators,[[qv: 29b,41a,64]] RAFT photopolymerization is an attractive option to (at least partially) decouple the polymerization temperature from the radical initiation process. RAFT photopolymerization may therefore be used to study the effect of temperature on the polymerization process. For example, Tan et al. extended their work on low temperature Photo‐PISA[[qv: 6b]] to study the effect of variable temperature on nanoparticle evolution during the Photo‐PISA process.^65^ As discussed above, near quantitative monomer conversions were observed within 30 min in the photoinitiated system even when conducted at room temperature due to rapid radical generation by SPTP under violet LED light. In comparison, initiation with ACVA at 70 °C was relatively slow requiring ~2 hours to reach high monomer conversion. Importantly, a similar degree of polymerization control was observed in both cases with typical dispersities of about 1.3 throughout the polymerization. Since the photodegradation behaviour of SPTP was deemed to be relatively insensitive to temperature, the effect of temperature on morphological evolution could be studied since similar polymerization rates could be obtained at the temperatures studied. Interestingly, through the construction of phase diagrams it was determined that as the temperature increased, the formation of higher order morphologies was favoured (**Figure** **4**). For example, a given formulation (DP = 200, solids content = 20 wt %) performed at 70 °C yielded a pure WLM phase but yielded only a mixture of morphologies when the polymerization was performed at lower temperatures. Given that similar molecular weight distributions were achieved, this result suggests the importance of reaction temperature on the PISA process particularly when conducted using monomer/polymer combinations with significant temperature dependent solubility.

### 4.2 Visible Light Mediated PISA without Exogenous Catalysts or Initiators

It is well known that UV light can be used to directly activate a RAFT type polymerization by causing photolytic cleavage of C–S bond yielding carbon centred radicals for initiating polymerization. This approach is attractive since it removes the need for exogenous catalysts/initiators to be added to initiate polymerization and has been studied intensively by a number of groups.[[qv: 33c,35d,66]] However, the use of UV light generally results in limited control over the polymerization particularly at high conversion owing to gradual degradation of the RAFT agent. Recently, research by our group,[[qv: 36c]] Qiao's group[[qv: 36d,45b,67]] and others[[qv: 45d,68]] has suggested that visible light can instead be used to directly activate the RAFT agent (without exogenous catalysts or initiators) leading to a higher degree of livingness compared to UV initiated approaches. This process is possible due to the weak n to π* absorption of some thiocarbonylthio species in the visible spectrum and has been proposed to proceed according to the photoiniferter (*photo*‐*ini*tiator‐trans*fer* agent‐*ter*minator) mechanism as originally proposed by Otsu.^69^ This visible light initiated polymerization depends strongly on both the type of RAFT agent employed as well as the effective light intensity. For example, we found that 4‐cyano‐4‐((dodecylsulfanylthiocarbonyl)sulfanyl)pentanoic acid (CDTPA) could be rapidly activated under blue light and this RAFT agent could mediate polymerization of MMA under blue light to high conversion within a few hours.[[qv: 36c]] However, Qiao's group reported that 2‐(*n*‐butyltrithiocarbonate) propionic acid (BTPA) appears to require a much longer reaction time for the polymerization of acrylates, (typically 24–48 h), as well as a relatively long inhibition period.[[qv: 36d,45b,67]]

**Figure 5:**
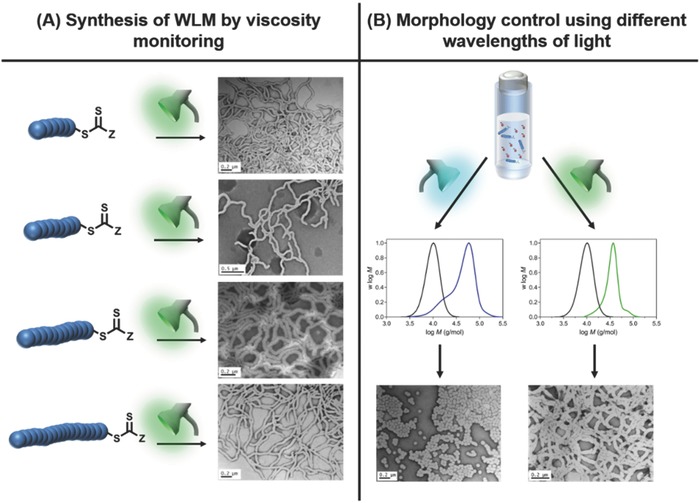
Visible light mediated PISA polymerization conducted in the absence of external catalyst or initiator. This process is used to demonstrate that (A) macroRAFT agents of different molecular weight can be used to synthesize WLM by viscosity monitoring and (B) the irradiation wavelength can control nanoparticle morphology due to differences in polymerization control. Adapted with permission.^24^ Copyright 2016, American Chemical Society.

We employed this technique to demonstrate the formation of various morphologies (S, WLM and V) under either visible blue (λ_max_ = 460 nm, 0.7 mW cm^−2^) or green light (λ_max_ = 530 nm, 0.7 mW cm^−2^) using a PISA approach.^24^ In this process the POEGMA macroRAFT agent (derived from CDTPA) acts simultaneously as a radical initiator, chain transfer agent and particle stabilizer in the dispersion polymerization of benzyl methacrylate (BzMA) in ethanolic conditions. Interestingly, the formation of WLM phases could be monitored in situ by observing increases in the viscosity of the polymerization mixture which is associated with inter‐worm entanglements. In comparison, this behaviour is typically only observed in a thermally initiated PISA polymerization of BzMA after the polymerization mixture has been quenched and cooled to room temperature which prevents in situ monitoring of the reaction viscosity. This behaviour is an advantage of these facile room temperature PISA polymerizations since it allows for the elusive intermediate WLM phase to be more reproducibly identified. Interestingly, when using macroRAFT agents of different molecular weight (from 7 200 to 10 300 g mol^–1^), the isolation of WLM could still be obtained by this method of viscosity monitoring suggesting its use as a metric to identify the WLM phase prior to conventional TEM analysis (**Figure** **5**A). As the molecular weight of the macroRAFT agent was increased, higher BzMA conversions were required to reach high viscosity. Finally, a number of formulations were identified in which morphology could be influenced by the wavelength of light used to initiate the polymerization. For example, a formulation irradiated under green light yielded WLM but the same formulation under blue light resulted in the formation of purely spherical particles. This unexpected result was attributed to the differing degrees of polymerization control under blue and green light (Figure 5B). This process suggests the feasibility of using extrinsically controlled parameters to modulate the nanoparticle morphology which is currently not achievable in a thermally‐initiated system.

Although not demonstrated in this work, one possible advantage of this system lies in its general applicability to different solvent systems since there is no need to solubilize a small molecule initiator/catalyst; the macroRAFT must be solvent soluble since it acts as the nanoparticle stabilizer. In addition, there is no need to remove residual photoinitiator or catalyst species that might be a potential source of toxicity. However, it should be noted that although high monomer conversion can be achieved in these catalyst/initiator free photopolymerization systems, the rates of polymerization are generally low compared to that reported with photoinitiator or photocatalyst species.^65^ Furthermore, these polymerizations generally require careful deoxygenation since even low concentrations of oxygen can quench the activated RAFT agent causing deviations from ideal polymerization behaviour.

During the revision of this manuscript, O'Reilly's group also reported a Photo‐PISA study in which the effect of different initiation methods on nanoparticle morphology was studied.^70^ In this work, the Photo‐PISA of HPMA was conducted in the absence of catalyst/initiator with radical generation instead occurring due to the photoiniferter mechanism. Interestingly, the authors found that the phase diagrams generated using either Photo‐PISA or a thermally initiated PISA approach differed significantly even when performed at the same reaction temperature. This behaviour was demonstrated to be due to differences in both the polymerization rate and end group fidelity between the two techniques.

### 4.3 Visible Light Mediated Photocatalysts for Conducting a PISA Process

**Figure 6:**
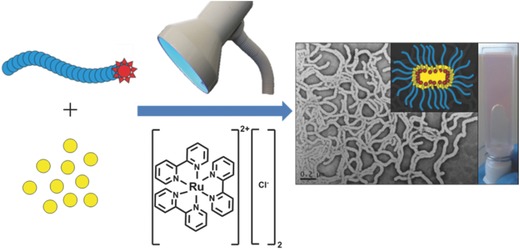
Application of blue visible light to mediate a PET‐RAFT dispersion polymerization in the presence of [Ru(bpy)_3_]Cl_2_. The formation of WLM can be monitored by in situ gelation of the reaction mixture. Reproduced with permission.[[qv: 2f]] Copyright 2015, American Chemical Society.

In 2014, our group demonstrated that photoredox catalysts such as Ir(ppy)_3_ and [Ru(bpy)_3_]Cl_2_ (at ppm concentrations) can be used to directly activate RAFT polymerization under visible light. This process was named photoinduced electron/energy transfer – reversible addition‐fragmentation chain transfer (PET‐RAFT) and proposed to proceed according to the mechanism provided in Figure 1. This process has been demonstrated to be compatible with a range of solvent and monomer systems and under both homogenous[[qv: 29a,36b,71]] and hetereogenous conditions.[[qv: 2f,72]] In addition, the photocatalyst can be used at much lower concentrations compared to traditional radical initiators, and generally provides a greater degree of polymerization control compared to catalyst and initiator‐free photoiniferter type polymerizations. In 2015, we used the PET‐RAFT system to demonstrate the first example of a visible light mediated PISA polymerization capable of yielding different morphologies (**Figure** **6**). In these initial studies, the ethanolic dispersion polymerization of BzMA under blue LED light (λ_max_ = 465 nm, 0.7 mW cm^−2^) was studied using [Ru(bpy)_3_]Cl_2_ as a photoredox catalyst owing to its excellent solubility in polar solvents.[[qv: 2f,73]] Although the room temperature polymerization was relatively slow (α ≈ 70% in 24 h), the formation of different morphologies (S, WLM and V) could be observed by varying a combination of solvent quality (MeCN:EtOH mixtures) and total solids content. The addition of MeCN was hypothesised to aid in morphological transformation by plasticising the core forming polymer and enabling micelle fusion into WLM.[[qv: 1s]] As in the visible light mediated photoiniferter system described above, the formation of WLM could be readily monitored at intermediate conversions due to the in situ viscous transition caused by worm entanglements. Furthermore, the photopolymerization could be temporally controlled by turning the LED source ON and OFF providing an additional means of synthetic control over the nanoparticle morphology. Such a fine degree of ON/OFF control over nanoparticle morphology is not possible under the typical conditions of a thermally initiated PISA process.

Szymański and Pérez‐Mercader utilized a similar approach for the aqueous polymerization of HPMA under blue LED light. Owing to the intrinsic fluorescent properties of the [Ru(bpy)_3_]Cl_2_ photoredox catalyst, the phase separation behaviour of this system could be readily studied using fluorescence microscopy.^74^ Interestingly, micron‐sized giant vesicles synthesized using this approach displayed some unusual light mediated growth and collapse behaviour.^75^

As an alternative to heavy‐metal based photoredox catalysts, Pan and coworkers performed PET‐RAFT polymerization using the organic dye, 10‐phenylphenothiazine (PTH) under PISA conditions.^76^ PTH has previously been implemented to perform either metal‐free ATRP^77^ or RAFT[[qv: 36b,71]] polymerization and enables the synthesis of well‐defined polymers without the use of heavy metals which may present undesirable toxicity. Although relatively high PTH concentrations were required, good control over the dispersion polymerization of BzMA was demonstrated and high monomer conversion was achievable within 33 h (α > 90%). Importantly, this metal free process enabled the synthesis of nanoparticles of different morphologies (S, WLM and V) and ON/OFF control over the polymerization was observed.

**Figure 7:**
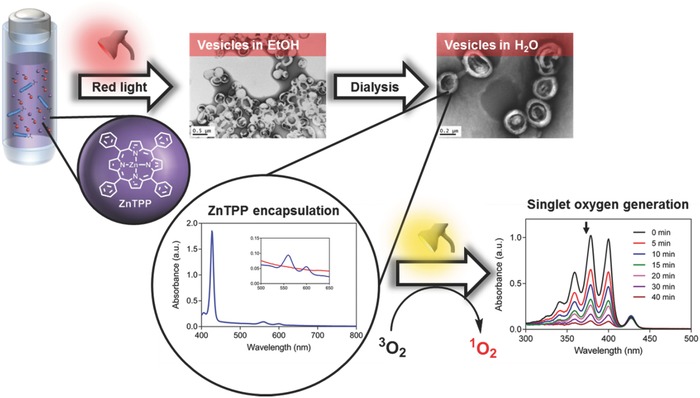
ZnTPP can be used as both a polymerization catalyst and light activated drug for photodynamic therapy (when encapsulated inside polymeric nanoparticles). Adapted with permission.[[qv: 72b]] Copyright 2016, American Chemical Society.

More recently, we demonstrated that Photo‐PISA could be extended to longer visible light wavelengths (red and yellow light) by the addition of the metalloporphyrin, 5,10,15,20‐tetraphenyl‐21H,23H‐porphine zinc (ZnTPP) which can activate the dispersion polymerization of benzyl methacrylate under low energy red light (λ_max_ = 635 nm) according to a PET‐RAFT mechanism.[[qv: 72b]] The use of longer wavelengths of visible light is advantageous due to the lower degree of nanoparticle induced scattering during the polymerization and potential for decreased side reactions particularly in the presence of sensitive moieties such as drugs or proteins. Furthermore, initiation using different wavelengths of light opens the possibility of utilising monomers with distinct absorptions in the visible spectrum. As in our previous studies on blue and green light mediated dispersion polymerization,^24^ nanoparticles of different morphologies (S, WLM, V) could be formed by manipulation of the target DP of BzMA, total solids content and solvent composition. Since ZnTPP acts catalytically during the polymerization and possess strongly hydrophobic character, we observed the encapsulation of the photocatalyst upon transferring the nanoparticles into water (**Figure** **7**). Interestingly, the ZnTPP encapsulated within the hydrophobic core of the nanoparticles could be further activated under visible light to generate singlet oxygen suggesting a dual use of ZnTPP as both a polymerization catalyst and light triggered drug for photodynamic therapy (Figure 7). Furthermore, the addition of ascorbic acid to the initial polymerization mixture was found to allow the initial photopolymerization to proceed without prior deoxygenation thereby simplifying the reaction setup. The trapping of photosensitized oxygen by ascorbic acid allowed for in situ chemical deoxygenation to be performed without specialized equipment (vacuum pump, inert gas etc.) and without inhibiting the generation of different nanoparticle morphologies. More recently, we have also demonstrated that in situ deoxygenation can be performed in the presence of other singlet oxygen quenchers such as 9,10‐dimethylanthracene (DMA). Replacing ascorbic acid with DMA allows for significantly faster kinetics (and a much shorter induction period) to be achieved whilst still allowing facile access to the full range of nanoparticle morphologies.^78^

**Figure 8:**
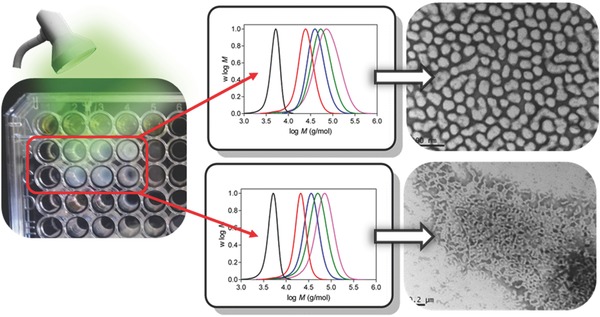
Oxygen tolerant photopolymerization can be used to perform aqueous RAFT dispersion polymerization in ultralow reaction volumes and in parallel under mild reaction conditions. Adapted with permission.^81^ Copyright 2017, The Royal Society of Chemistry.

The ability to perform polymerization in a non‐deoxygenated vessel opens up the possibility of performing synthesis in ultralow volumes in non‐traditional reactor systems.^79^ For example, Chapman et al. demonstrated that oxygen tolerant RAFT polymerization enables polymer syntheses to be carried out in microtitre plates at 40 µL volumes.^79^ These low volume syntheses enable the high throughput syntheses of polymer libraries without specialized equipment such as glove boxes and automated synthesizers.^80^ When applied to RAFT dispersion polymerization, such an approach is also likely to be useful for the high throughput optimization of reaction parameters particularly when investigating the formation of different morphologies. We recently developed an organic dye/reducing agent photoinitiation system to perform oxygen tolerant RAFT photopolymerization in very low reaction volumes (> 20 µL) under green LED light (λ = 530 nm, 2.65 mW cm^−2^).^81^ The radical generation process was found to be inherently tolerant to molecular oxygen due to the photocatalytic reduction of oxygen by eosin Y into hydrogen peroxide which is subsequently reduced into initiating hydroxyl radicals. This process was sufficiently tolerant to oxygen to enable the benchtop synthesis of well‐defined RAFT polymers in discrete droplets or microtiter plates without specialized equipment. We used this oxygen tolerant photoinitiation process to perform aqueous RAFT dispersion polymerization of DAAm according to a PISA approach (**Figure** **8**). These photopolymerizations were rapid with near quantitative monomer conversion (> 98%) achievable within 4 h of green light irradiation. By varying the target DP of DAAM, different morphologies such as S and WLM could be formed at ultralow volumes (> 50 µL). Furthermore, performing these polymerizations in a 96 well microtiter plate enabled multiple syntheses to be performed in parallel suggesting the possibility of performing high throughput syntheses for the production of phase diagrams. The preliminary results of this study suggest the possibility of using visible light mediated oxygen tolerant polymerization to perform high throughput screening of the synthetic aspects of the PISA process. Furthermore, a nanoparticle library presenting different sizes and morphologies may be useful for performing systematic studies in applications such as drug delivery.

## 5 Future Perspective and Challenges

The application of the PISA technique is generally favoured for its ease of setup along with the ability to produce different nanoparticle morphologies at high solids content. Such a process is much more amenable to scale up especially compared to conventional self‐assembly techniques. However, the use of visible light or UV light to initiate polymerization (in either homogenous or heterogenous conditions), poses a number of challenges in terms of scale compared to thermally or redox initiated systems. In particular, the attenuation of UV or visible light through the reaction mixture (even at the laboratory scale), can induce a strong dependence of the polymerization rate/kinetics on the geometry, size and materials of the vessel itself.^82^ Furthermore, the formation of light intensity gradients may introduce heterogeneity into an imperfectly stirred system. In a general CLRP process, this is likely to result in broader molecular weight distributions.

In a dispersion polymerization, the role of light scattering on the dynamic polymerization rate is likely to be more complex than that observed under homogenous conditions. Since Rayleigh scattering increases proportionally to d^6^/λ^4^, it is likely that the effects of light scattering will become more apparent as the nanoparticles increase in size (and change morphology) during the polymerization.^83^ One approach to overcome this limitation has already been demonstrated in Photo‐PISA by using longer wavelengths of light (towards the red region) to control the polymerization and thereby lower the amount of nanoparticle‐induced scattering.[[qv: 72b]] However, to date, the effect of wavelength on a Photo‐PISA process has not been systematically studied. Furthermore, a comprehensive study on the effect of incident light scattering and absorption during the PISA process would be beneficial.

**Figure 9:**
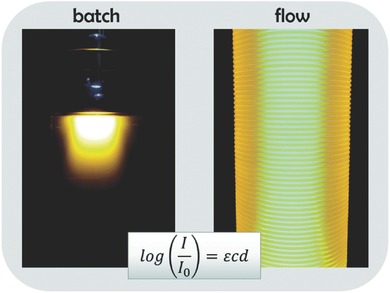
Difference in light penetration through batch and flow reactors. Reproduced with permission.^88^ Copyright 2015, The Royal Society of Chemistry.

As an alternative to traditional batch reactors, the high surface area to volume ratio offered by continuous flow reactors is attractive for overcoming issues of scale in Photo‐PISA. In addition, the distribution of light intensity throughout a flow reactor is essentially uniform minimising the presence of local inhomogeneities (**Figure** **9**). Work by Junkers,^84^ Johnson,^85^ Hawker,^86^ and our group^87^ have demonstrated a number of advantages of applying photoinitiated CLRP in flow reactors particularly with regards to reproducibility and the increase in polymerization rate compared to batch reactors.

This flow polymerization setup was recently studied in a thermally initiated PISA system and is likely to have significant benefits for Photo‐PISA systems particularly in terms of production rates and uniform light penetration.^89^ Apart from generating nanomaterials at significantly higher throughput, faster polymerization rates under both homogenous and heterogeneous conditions would be expected compared to traditional batch reactors.^88^

Some of the earliest work on photopolymerization in CLRP systems demonstrated the ability to temporally control the polymerization by modulation of the light source.^90^ In a Photo‐PISA system, the polymerization can be readily turned ON/OFF facilitating mechanistic studies on the evolution of nanoparticle morphology during the polymerization. Furthermore, the polymerization rates are generally fast (when using a photoinitiator) compared to thermally initiated PISA and can be finely controlled by changing the light intensity. The (partial) decoupling of the polymerization temperature and initiation rate should also allow for a more systematic study on the role of polymerization rate and temperature in determining the final nanoparticle morphology. Although some thermally initiated PISA studies have implicated a link between the polymerization rate and morphology, different polymerization temperatures or initiator ratios were required.[[qv: 1u]] In contrast, photopolymerization rates are for the most part highly sensitive to the irradiation intensity and so can be used to more systematically study the effect of polymerization rate (and temperature) on morphological evolution.^65^ Further studies on additional monomer/solvent systems are likely to have important implications regarding the reproducibility of PISA polymerizations.

The facile conditions of visible light photopolymerization have proven to be highly compatible with biologically active species such as proteins or drugs. It should be noted that low temperature thermal initiators also enable mild polymerization conditions to be achieved, however the rate of radical generation is generally slow and highly sensitive to temperature unlike most photoinitiation systems. Using a Photo‐PISA approach, the encapsulation of BSA as a model protein has been demonstrated along with its subsequent triggered release under a carbon dioxide stimulus.^62^ This approach is particularly promising for the production of polymer‐protein conjugates without the need for covalent modification of the protein substrate. Such polymer‐protein conjugates have also been demonstrated to possess significantly improved stability relative to the native protein.^91^ Further work needs to be done on methods to improve the protein/drug loading efficiency of the Photo‐PISA process since the lumen encapsulation process is relatively inefficient and significant purification of the protein loaded vesicles is currently required. Improving the encapsulation efficiencies of such systems is particularly important due to the generally high cost of protein based therapeutics. Interestingly, recent work by Armes' group has demonstrated that biodegradable polymeric nanoparticles can be synthesized using a PISA approach. By incorporating monomers capable of undergoing radical ring opening polymerization such as cyclic allylic sulfides, degradation of the polymer backbone can be realised under reducing conditions.[[qv: 12a]] Finally, in order to apply PISA derived nanoparticles in a biological setting, the biocompatibility and long‐term stability of these nanoparticles still needs to be determined.

Finally, the oxygen tolerant nature of some visible light mediated polymerizations can be applied to perform PISA synthesis without specialized equipment (vacuum pump, inert gas etc.). This process can allow for nanoparticle synthesis to be performed in ultralow volumes and high throughput which lends itself to the more efficient production of PISA phase diagrams which can require extensive experimentation time and resources to produce. In addition, the simplifying of a typical polymerization setup should enable more facile access of the PISA technique (and all its associated advantages) to non‐experts in the field.

## Conflict of Interest

The authors declare no conflict of interest.
